# Goal-directed fluid restriction during brain surgery: a prospective randomized controlled trial

**DOI:** 10.1186/s13613-017-0239-8

**Published:** 2017-02-16

**Authors:** Jinfeng Luo, Jing Xue, Jin Liu, Bin Liu, Li Liu, Guo Chen

**Affiliations:** Department of Anesthesiology and Translational Neuroscience Center, West China Hospital, Sichuan University, No. 37 Guoxue Xiang, Wuhou District, Chengdu, 610041 Sichuan People’s Republic of China

**Keywords:** Fluid management, Goal-directed therapy, Goal-directed fluid restriction, Brain surgery, Postoperative complications, ICU length of stay

## Abstract

**Background:**

The value of goal-directed fluid therapy in neurosurgical patients, where brain swelling is a major concern, is unknown. The aim of our study was to evaluate the effect of an intraoperative goal-directed fluid restriction (GDFR) strategy on the postoperative outcome of high-risk patients undergoing brain surgery.

**Methods:**

High-risk patients undergoing brain surgery were randomly assigned to a usual care group (control group) or a GDFR group. In the GDFR group, (1) fluid maintenance was restricted to 3 ml/kg/h of a crystalloid solution and (2) colloid boluses were allowed only in case of hypotension associated with a low cardiac index and a high stroke volume variation. The primary outcome variable was ICU length of stay, and secondary outcomes were lactates at the end of surgery, postoperative complications, hospital length of stay, mortality at day 30, and costs.

**Results:**

A total of 73 patients from the GDFR group were compared with 72 patients from the control group. Before surgery, the two groups were comparable. During surgery, the GDFR group received less colloid (1.9 ± 1.1 vs. 3.9 ± 1.6 ml/kg/h, *p* = 0.021) and less crystalloid (3 ± 0 vs. 5.0 ± 2.8 ml/kg/h, *p* < 0.001) than the control group. ICU length of stay was shorter (3 days [1–5] vs. 6 days [3–11], *p* = 0.001) and ICU costs were lower in the GDFR group. The total number of complications (46 vs. 99, *p* = 0.043) and the proportion of patients who developed one or more complications (19.2 vs. 34.7%, *p* = 0.034) were smaller in the GDFR group. Hospital length of stay and costs, as well as mortality at 30 day, were not significantly reduced.

**Conclusion:**

In high-risk patients undergoing brain surgery, intraoperative GDFR was associated with a reduction in ICU length of stay and costs, and a decrease in postoperative morbidity.

*Trial registration* Chinese Clinical Trial Registry ChiCTR-TRC-13003583, Registered 20 Aug, 2013

## Background

Perioperative fluid administration has been shown to be a major determinant of postoperative outcome [1]. The amount of fluid administered during the perioperative period depends on multiple factors such as preoperative hydration, intraoperative blood loss, hemodynamic stability, as well as habits and believes of anesthesiologists and surgeons [2]. Fluid needs may be highly variable from one patient to the other and are hardly predictable from classical physiological parameters such as heart rate, blood pressure, and central venous pressure. Fluid titration based on the measurements of advanced hemodynamic parameters, such as cardiac output and dynamic predictors of fluid responsiveness, has been shown to be useful to improve the outcome of patients undergoing major abdominal, vascular, and orthopedic procedures [3–6].

Whether such strategies may also be useful in patients undergoing brain surgery is unknown. In this specific context, the temptation is to keep patients as dry as possible to prevent brain swelling. However, blinded or uncontrolled fluid restriction may expose patients to hypovolemia-related complications [1]. We hypothesized that advanced hemodynamic measurements may be useful to objectively balance the risks of fluid restriction (hypovolemia, hypotension, and cerebral ischemia) with the risk of inducing or worsening cerebral edema [7, 8]. In line with this concept, we designed an intraoperative fluid management protocol where minimal fluid maintenance with a crystalloid solution (fluid restriction) was combined with the administration of fluid boluses in case of severe and documented hypovolemia.

The goal of the present study was therefore to assess the effects of an intraoperative goal-directed fluid restriction (GDFR) strategy on the postoperative outcome of high-risk patients undergoing brain surgery.

## Methods

### Patient selection

Adult patients undergoing elective craniotomy for brain tumor resection, brain abscess, or intracranial aneurysm were considered for enrollment. Inclusion criteria were age >18, ASA score III or IV, and expected duration of surgery >2 h. Patients with a body weight <40 kg or >100 kg were excluded, as well as patients with cardiac arrhythmia (well-known limitation to the use of the stroke volume variation as an indicator of fluid responsiveness) [9]. The study was approved by the Ethical Committee of the West China Hospital from Sichuan University (No. 2012-104). After obtaining written informed consent, patients were randomly assigned to a standard fluid management group (Control) or to a GDFR group. A random number table was used to generate the random number sequence. All random numbers were concealed in sealed envelopes and assigned to a patient when entering the operating room.

### Intraoperative monitoring and management

In addition to pulse oximetry, capnography, and heart rate monitoring, all patients had a radial arterial line in place for continuous blood pressure monitoring and a BIS sensor in place to monitor depth of anesthesia. Tidal volume was set at 8 ml/kg and respiratory frequency was adapted to maintain end-tidal CO_2_ between 30 and 35 mmHg. Anesthesia induction was done with propofol (2 mg/kg) and rocuronium (1 mg/kg), and then propofol and remifentanil were used to maintain depth of anesthesia (BIS in the range 40–60). Body temperature was maintained close to normal using warmed solutions and insulation blankets. Per our neurosurgical policy, all patients received mannitol the day before surgery (250 ml), at the beginning of the surgical procedure (250 ml), and the day after surgery (125 ml).

In the control group, no recommendation was given for fluid and hemodynamic management during and after surgery, and therapeutic decisions were left at the discretion of the attending anesthesiologist and intensivist. In the GDFR group and during surgery, fluid maintenance was set at 3 ml/kg/h of normal saline with an infusion pump, and additional colloid (gelatins or hydroxyethyl starches) boluses (200 ml) were allowed only in case of systemic hypotension (MAP < 65 mmHg) with a cardiac index (CI) <2.5 l/min/m^2^ and a stroke volume variation (SVV) >15%. In case of hypotension with a CI >2.5, the recommendation was to give a vasopressor. If CI was <2.5 and SVV <15%, the recommendation was to give an inotrope. The FloTrac/Vigileo system (Edwards Lifesciences, Irvine, CA) was used to continuously monitor CI and SVV.

### Outcome variables

The primary outcome variable was ICU length of stay. Secondary outcome variables were lactates at the end of surgery, postoperative complications at day 30, postoperative morbidity (the proportion of patients who developed one or more complications) at day 30, mortality at day 30, hospital length of stay, and costs.

### Statistical analysis

Median ICU length of stay was 5 ± 1.6 days in our institution, and we assumed that GDFR may decrease it by 1 day or 20%. A study sample size of 60 patients in each group was calculated for two-sided tests with type I error of 5% and power of 90%. Owing to an anticipated loss of several patients entering the study, we planned to include 75 patients in each group. For a test of normal distribution, the Kolmogorov–Smirnov test was used. Continuous data with normal distribution were tested with paired or unpaired *t* tests, non-normally distributed data using Mann–Whitney U test and Wilcoxon rank-sum test for unpaired and paired results, respectively. Changes in lactate over time were tested using analysis of variance (ANOVA) on repeated measurements. Categorical data were tested using Chi-square test and Chi-square test for trend. Data are presented as mean ± standard deviation when normally distributed and as median [interquartile ranges] in case of abnormal distribution. A *p* < 0.05 was considered statistically significant for all tests. All calculations were done with MedCalc^®^ version 10.4.8.0 (MedCalc Software, Mariakerke, Belgium).

## Results

A total of 150 patients were randomized (75 in each group). Despite preoperative approval, five patients refused to participate in the study after surgery and had to be excluded from the analysis. Thus, 73 patients from the GDFR group were compared to 72 patients from the control group.

### Baseline and surgical characteristics

Reasons for surgery were brain tumor (n = 87), intracranial aneurysm (n = 55), or brain abscess (n = 3) and were well balanced between the two groups (Table 1). The GDFR group and the control group were comparable in terms of age, comorbidities, ASA score, and POSSUM score (Table 1). Surgery duration was comparable in both groups as well as intraoperative estimated blood loss (Table 2). During surgery, the GDFR group received significantly less colloid and crystalloid than the control group (Table 2).

**Table 1:** Preoperative characteristics of the study population

	GDFR group (n = 73)	Control group (n = 72)	*p* value
Male/female	30:43	32:40	0.738
Age	61 ± 13	62 ± 13	0.693
Age ≥ 70 years	20	24	0.474
Weight (kg)	59 ± 11	60 ± 12	0.417
Height (cm)	160 ± 8	162 ± 7	0.105
POSSUM (physiology score)	21 ± 7	23 ± 6	0.151
POSSUM (operative score)	13 ± 4	13 ± 3	0.394
ASA (3:4)	70:3	70:2	1.000
Comorbidities			
Coronary artery disease	5	7	0.563
Hypertension	45	37	0.243
Peripheral artery disease	0	0	1.000
COPD	11	18	0.151
Other pulmonary pathology	4	5	0.745
Cerebrovascular disease	26	29	0.610
Diabetes mellitus	10	11	0.818
Chronic kidney disease	1	1	0.992
Malignancy	33	30	0.738
Diagnosis			
Intracranial aneurysm	26	29	0.610
Intracranial mass	46	41	0.500
Brain abscess	1	2	0.620

Values are presented as mean ± standard deviation

*POSSUM* physiological and operative severity score for the enumeration of mortality and morbidity, *COPD* chronic obstructive pulmonary disease

**Table 2:** Patient characteristics during and 24 h after surgery

	GDFR group (n = 73)	Control group (n = 72)	*p* value
Patient position			
Supine position (n)	41	45	0.500
Left-lateral position (n)	18	14	0.549
Right-lateral position (n)	12	10	0.818
Prone position (n)	2	3	0.679
Beginning of surgery			
CI (ml/min/m^2^)	3 ± 1.2	NA	NA
SVV (%)	12 ± 5	NA	NA
During surgery			
Crystalloids (ml/kg/h)	3 ± 0	5.0 ± 2.8	<0.001
Colloids (ml/kg/h)	1.9 ± 1.1	3.9 ± 1.6	0.021
Colloids (ml)	563 ± 550	1050 ± 548	<0.001
Colloids (nb of bolus per patient)	3 ± 3	5 ± 3	<0.001
Autologous blood transfusion (ml)	n = 5, 280 ± 109	n = 14, 375 ± 198	0.224
Red blood cell (ml)	n = 3, 400 ± 200	n = 2, 400 ± 282	0.089
Fresh frozen plasma (ml)	0	0	1
Estimated blood loss (ml)	287 ± 179	305 ± 273	0.535
Metaraminol (no. of patients)	39	18	<0.001
Ephedrine (no. of patients)	40	21	<0.001
Dopamine (no. of patients)	5	3	0.479
Dobutamine (no. of patients)	1	0	0.319
Norepinephrine (no. of patients)	3	3	0.981
End of surgery			
CI (ml/min/m^2^)	3.6 ± 0.7	NA	NA
SVV (%)	7 ± 2	NA	NA
Length of surgery (min)	274 ± 72	276 ± 66	0.864
Dopamine (n)	1	0	0.319
Norepinephrine (n)	0	1	0.312
24 h after surgery			
Crystalloids (ml/kg/h)	2.6 ± 1.3	2.6 ± 0.8	0.881
Colloids (ml/kg/h)	0.2 ± 0.3	0.2 ± 0.3	0.868
Blood (ml/kg/h)	0	0	0.746
Fresh frozen plasma (ml)	0	0	0.744
Estimated blood loss (ml)	98 ± 65	115 ± 99	0.206
Norepinephrine (n)	3	3	0.981
Dopamine (n)	5	3	0.479
Vasodilatation therapy (n)	5	11	0.105
Diuretic support (n)	45	38	0.281

Values are presented as mean ± standard deviation

*CI* cardiac index, *HR* heart rate, *MAP* mean arterial pressure, *N/A* not applicable, *SVV* stroke volume variation

### Outcome variables

**Fig. 1:**
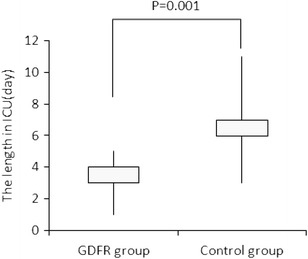
Length of stay in the intensive care unit (ICU) in the goal-directed fluid restriction (GDFR) and in the control groups

The ICU length of stay was significantly shorter in the GDFR group (3 days [1–5] vs. 6 days [3–11], *p* = 0.001) (Fig. 1). At the end of the surgical procedure, lactates were lower in the GDFR group (1.79 ± 0.85 vs. 2.23 ± 1.36 mmol/l, *p* = 0.003). The total number of complications and the proportion of patients who developed one or more postoperative complications were lower in the GDFR group, as well as ICU costs (Table 3). Median hospital length of stay was decreased by 2 days, but the difference did not reach statistical significance (Table 3). No differences were found in heart rate, mean arterial pressure, P_ET_CO_2_, natremia, and urine output during the surgery between the two groups (Table 4).

**Table 3:** Postoperative outcome

Parameters	GDFR group (n = 73)	Control group (n = 72)	*p* value
ICU length of stay (days)	3 (1–5)	6 (3–11)	0.001
Mortality (n/%)	4/5.5	9/12.5	0.158
Hospital length of stay (days)	15 (7–23)	17 (9–27)	0.069
Complications (day 30)			
Total (n)	46	99	0.043
Patients with one or more complications (n/%)	14/19.2	25/34.7	0.034
Vomiting (n/%)	2/2.7	2/2.8	0.989
Coma (n/%)	3/4.1	2/2.8	0.660
Seizure (n/%)	1/1.4	3/4.2	0.304
Encephaledema (CT/MRI) (n/%)	9/12.3	16/22.2	0.115
Pneumonia (n/%)	9/12.3	20/27.8	0.020
Sepsis (n/%)	0/0	2/2.8	0.152
Ventilator support (n/%)	6/8.2	12/16.7	0.123
New onset of ventilator support (n/%)	3/4.1	8/11.1	0.111
AKI (without dialysis) (n/%)	3/4.1	5/6.9	0.455
Renal failure with dialysis (n/%)	0/0	1/1.4	0.312
Stroke (including TIA) (n/%)	1/1.4	0/0	0.319
Deep vein thrombosis (n/%)	0/0	4/5.6	0.041
Graft thrombosis (n/%)	0/0	1/1.4	0.312
Wound infection/dehiscence (n/%)	0/0	0/0	1
Skin lesions (n/%)	8/11.0	16/22.2	0.068
Arrhythmias (non-life threatening) (n/%)	1/1.4	2/2.8	0.552
Arrhythmias (life threatening) (n/%)	0/0	1/1.4	0.312
Heart failure/pulmonary edema (n/%)	0/0	4/5.6	0.041
Acute myocardial infarction (n/%)	0/0	0/0	1
Pulmonary embolism (n/%)	0/0	0/0	1
Costs			
Hospital costs ($)	9218 ± 2890	10,249 ± 3175	0.482
ICU costs ($)	1776 ± 459	3080 ± 700	0.037

Values are presented as mean ± standard deviation

*ICU* intensive care unit, *AKI* acute kidney injury

**Table 4:** Patient characteristics during surgery

Variable	Beginning of surgery	1 h	2 h	3 h	4 h	End of surgery
Number						
GDFR group	73 (100%)	73 (100%)	73 (100%)	36 (49%)	25 (34%)	73 (100%)
Control group	72 (100%)	72 (100%)	72 (100%)	37 (51%)	24 (33%)	72 (100%)
*p* value	1.00	1.00	1.00	0.80	0.92	1.00
MAP (mmHg)						
GDFR group	83 ± 14	75 ± 9	77 ± 10	77 ± 11	73 ± 9	82 ± 11
Control group	80 ± 15	78 ± 11	80 ± 14	76 ± 11	82 ± 11	78 ± 11
*p* value	0.17	0.09	0.16	0.93	0.12	0.07
P_ET_CO_2_ (mmHg)						
GDFR group	29 ± 3	29 ± 4	29 ± 3	29 ± 3	29 ± 3	31 ± 4
Control group	30 ± 3	29 ± 3	29 ± 3	30 ± 3	32 ± 4	28 ± 3
*p* value	0.20	0.68	0.69	0.44	0.06	0.79
Sodium (mmol/l)						
GDFR group	137.8 ± 4.8	134.5 ± 5.7	135.9 ± 4.7	136.8 ± 5.5	136.2 ± 3.3	137.3 ± 4.5
Control group	136.0 ± 5.5	134.3 ± 5.8	136.1 ± 5.2	137.4 ± 5.3	139.0 ± 6.7	137.5 ± 5.1
*p* value	0.03	0.81	0.86	0.64	0.07	0.84
Diuresis (ml/kg/h)						
GDFR group	0	5.58 ± 1.74	5.19 ± 1.37	4.97 ± 1.85	4.41 ± 1.73	4.12 ± 1.39
Control group	0	5.76 ± 1.50	5.52 ± 1.77	5.03 ± 1.63	5.01 ± 1.45	4.12 ± 1.39
*p* value	1	0.52	0.18	0.92	0.26	0.80

Values are presented as mean ± standard deviation

*MAP* mean arterial pressure, *P*_*ET*_ *CO*_*2*_ peak end-tidal CO_2_

## Discussion

In high-risk patients undergoing brain surgery, our study shows that the use of GDFR was associated with a decrease in fluid volumes, less postoperative complications, and a shorter ICU length of stay.

Many studies have investigated the value of goal-directed fluid therapy in patients undergoing major abdominal, vascular, and orthopedic surgeries [3–6]. Conflicting results have been reported, but recent meta-analysis [3–6] has suggested an overall reduction in postoperative morbidity around 25–50%, associated with a 1–2-day reduction in hospital length of stay. As far as we know, this is the first study investigating the effects of a goal-directed fluid strategy in patients undergoing brain surgery. The main objective of goal-directed strategies is to rationalize the way fluid is administered during the perioperative period [10]. A U-shaped relationship has been described between the perioperative fluid volume and postoperative complications [1]. Patients who do not receive enough fluid may develop complications related to hypovolemia, such as acute renal failure, myocardial injury, and cerebral ischemia. On the other hand, patients receiving too much fluid may develop complications associated with fluid overload, such as tissue edema, which may be responsible for prolonged mechanical ventilation and delayed wound healing [11].

For specific surgical procedures, such as pneumonectomy, liver resection, and neurosurgery, the temptation has always been to keep patients as dry as possible to prevent pulmonary edema, surgical bleeding, and brain swelling, respectively. However, blinded or uncontrolled fluid restriction may expose patients to hypovolemia-related complications [1, 12]. We hypothesized that advanced hemodynamic measurements may be useful to prevent both insufficient and excessive fluid management and improve postoperative outcome. We therefore designed an intraoperative fluid management protocol where minimal fluid maintenance with a crystalloid solution (fluid restriction) was combined with the administration of fluid boluses only in case of severe hypovolemia defined by the association of a low cardiac index with a high SVV. The SVV has been shown to be useful to predict fluid responsiveness in many different settings [13]. Pending limitations are respected [9]; SVV >10–13% identifies fluid responder patients with high sensitivity and specificity [13]. We decided to use a higher cutoff value to keep our patients on the “dry” side, but we also decided to allow fluid boluses when SVV was >15% to prevent excessive fluid restriction and hypovolemia-related complications. Goal-directed fluid strategies have the advantage to rationalize the way patients are treated [10]. However, we believe that fluid management protocols must be adapted to clinical and surgical situations. The use of high SVV target values (around 18–20%) has recently been proposed in patients undergoing liver resection to limit surgical bleeding as much as possible [14]. We thought it might be wise to adopt a similar strategy in neurosurgical patients to minimize cerebral edema. With our GDFR protocol, patients received less crystalloid and colloid than the control group, but did not develop more often hypovolemia-related complications such as acute kidney and myocardial injury.

Our study also showed a significant reduction in ICU costs with GDFR, which is logical when considering the observed reduction in ICU length of stay, likely related to the lower number of patients who developed complications (Table 3). Sadique et al. [15] recently reported the financial results of the large UK Optimise trial and showed that patients treated with goal-directed fluid therapy were on average £400 less expensive to treat than control patients. In patients undergoing head and neck surgery in the USA, in whom ICU length of stay was significantly reduced when using goal-directed fluid therapy, Hand et al. [16] recently reported savings exceeding $3000 per patient. Surgical and anesthesia costs in China are not comparable to those observed in the UK or in the USA. However, as suggested by previous studies [17, 18] our financial findings confirm that the improvement in postoperative outcome may offset the costs associated with the implementation of goal-directed fluid therapy.

Our study has several limitations. First, this is a single-center study where we compared a GDFR strategy to standard hemodynamic management at West China Hospital in Chengdu, Sichuan. We also focused on high-risk patients (ASA III & IV). Thus, our results may not be extrapolated to other institutions having different anesthesia and surgical practices, as well as to low-risk (ASA I & II) neurosurgical patients. Three patients had a brain abscess and did not require emergency surgery. Thus, they were considered as elective patients and enrolled in our study. We acknowledge their specificity but confirm that similar results were observed after excluding them from the analysis. Although based on physiological rational, the 15% cutoff value used for SVV was arbitrary and other studies would be useful to investigate whether better results could be observed when using lower (e.g., 13%) or higher (e.g., 18%) cutoff values. Also, we cannot draw any conclusion regarding the superiority of our original GDFR strategy over a more classical goal-directed fluid strategy (e.g., stroke volume optimization) or over fluid restriction alone. The reliability of the FloTrac/Vigileo system to measure cardiac output has been questioned when compared to clinical reference techniques such as pulmonary thermodilution and echocardiography [19]. Of note, most limitations have been described in patients with septic shock receiving vasopressors, or during liver transplantation [20]. Recent meta-analysis of validation studies suggests that both accuracy and precision are comparable to those observed with other continuous cardiac output monitoring techniques currently available on the market [21]. And many studies have demonstrated that FloTrac-derived SVV is an accurate predictor of fluid responsiveness; pending limitations are respected [20].

## Conclusion

In high-risk patients undergoing brain surgery, our intraoperative GDFR strategy was associated with a significant decrease in ICU length of stay, costs, and postoperative complications. Larger studies are needed to confirm our findings and assess the impact on hospital length of stay and hospital finances.

## Abbreviations

GDFR: goal-directed fluid restriction
ICU: intensive care unit
ASA: American Society of Anesthesiologists
CI: cardiac index
SVV: stroke volume variation
HR: heart rate
MAP: mean arterial pressure
N/A: not applicable
POSSUM: physiological and operative severity score for the enumeration of mortality and morbidity
